# Evaluation of Integrated Community Case Management in Eight Districts of Central Uganda

**DOI:** 10.1371/journal.pone.0134767

**Published:** 2015-08-12

**Authors:** Denis Mubiru, Robert Byabasheija, John Baptist Bwanika, Joslyn Edelstein Meier, Godfrey Magumba, Flavia Mpanga Kaggwa, Jackson Ojera Abusu, Alex Chono Opio, Charles Clarke Lodda, Jaanki Patel, Theresa Diaz

**Affiliations:** 1 Malaria Consortium, Kampala, Uganda; 2 Keeping Children and Mothers Alive, UNICEF Uganda, Kampala, Uganda; 3 Epidemiology and Health Policy & Practice, Columbia University Mailman School of Public Health, New York, New York, United States of America; 4 Health Section, Programme Division, UNICEF Headquarters, New York, New York, United States of America; Iran University of Medical Sciences, Islamic Republic of Iran

## Abstract

**Objective:**

Evidence is limited on whether Integrated Community Case Management (iCCM) improves treatment coverage of the top causes of childhood mortality (acute respiratory illnesses (ARI), diarrhoea and malaria). The coverage impact of iCCM in Central Uganda was evaluated.

**Methods:**

Between July 2010 and December 2012 a pre-post quasi-experimental study in eight districts with iCCM was conducted; 3 districts without iCCM served as controls. A two-stage household cluster survey at baseline (n = 1036 and 1042) and end line (n = 3890 and 3844) was done in the intervention and comparison groups respectively. Changes in treatment coverage and timeliness were assessed using difference in differences analysis (DID). Mortality impact was modelled using the Lives Saved Tool.

**Findings:**

5,586 Village Health Team members delivered 1,907,746 treatments to children under age five. Use of oral rehydration solution (ORS) and zinc treatment of diarrhoea increased in the intervention area, while there was a decrease in the comparison area (DID = 22.9, p = 0.001). Due to national stock-outs of amoxicillin, there was a decrease in antibiotic treatment for ARI in both areas; however, the decrease was significantly greater in the comparison area (DID = 5.18; p<0.001). There was a greater increase in Artemisinin Combination Therapy treatment for fever in the intervention areas than in the comparison area but this was not significant (DID = 1.57, p = 0.105). In the intervention area, timeliness of treatments for fever and ARI increased significantly higher in the intervention area than in the comparison area (DID = 2.12, p = 0.029 and 7.95, p<0.001, respectively). An estimated 106 lives were saved in the intervention area while 611 lives were lost in the comparison area.

**Conclusion:**

iCCM significantly increased treatment coverage for diarrhoea and fever, mitigated the effect of national stock outs of amoxicillin on ARI treatment, improved timeliness of treatments for fever and ARI and saved lives.

## Introduction

Pneumonia, diarrhoea and malaria are major causes of mortality in children under five years of age (U5) in Sub-Saharan Africa, including Uganda [1]. The correct treatment of these conditions is effective in reducing childhood mortality [2]. However, in most high-mortality countries, facility-based services are not meeting the need for timely treatment [3]. Although integrated community case management (iCCM), wherein appropriately trained community health workers (CHWs) treat uncomplicated pneumonia, diarrhoea and malaria, has been shown to be a feasible strategy to complement facility-based management for areas that lack access to health facilities, [4,5] the impact of this strategy has almost exclusively been evaluated in Asia, with only one evaluation of this strategy in sub-Saharan Africa demonstrating a mortality impact [6–11].

From 2002 to 2006, Uganda implemented Home Based Management of Fever (HBMF) nationwide. Village Health Teams (VHTs) comprised of four CHWs were trained to presumptively treat fever among children U5 with chloroquine and Fansidar (SP). In 2006, Uganda changed the first line treatment for malaria to Artemisinin-based Combination Therapies (ACT). In 2004, VHTs began managing diarrhoea with oral rehydration solution (ORS) and in 2007 zinc was added to the treatment protocol. In 2010, the Ugandan Ministry of Health (MOH) officially launched iCCM by adopting a policy to allow VHTs to treat acute respiratory infection (ARI), which was presumed to be pneumonia, with amoxicillin. Subsequently, VHTs could treat malaria with ACT, diarrhoea with ORS and zinc, and ARI with amoxicillin.

Malaria Consortium, supported by UNICEF, introduced iCCM in three districts (later re-configured as eight districts, as described below) in the Central Region of Uganda in July 2010. The program was evaluated after two years to determine whether provision of iCCM by VHTs led to significant changes in care seeking behaviour and treatment.

## Methods

### Study Design and Population

A pre-post quasi-experimental design with intervention and comparison areas was used to determine the impact of iCCM in the eight districts. Three intervention districts were selected based on the following: 1) a high poverty score, 2) a high expected absolute number of annual under five deaths, 3) existence of partners with iCCM capacity and 4) exclusion of districts that already had partners delivering iCCM. Between July 2010 and December 2012, iCCM was implemented in the selected three districts (Masaka, Mpigi and Wakiso) which in 2011 were divided into eight districts by the government of Uganda (Wakiso, Mpigi, Butambala, Gomba, Masaka, Lwengo, Bukomansimbi and Kalungu). The combined 2012 estimated population was 3,805,100.

Four VHT members per village were trained in the basic package to deliver or promote the use of preventive interventions, particularly hygiene, immunization, hand washing, optimal complementary feeding, insecticide treated nets and intermittent preventive treatment of malaria during pregnancy. iCCM adds treatment to the VHT preventive platform. For the iCCM component, 2 VHT members who ranked highest per village during the post-basic package training evaluation, were trained to use an algorithm for diagnosing conditions and taught how to dispense medications for treating children under 5 years of age who had an ARI (diagnosed through cough and fast breathing and presumed to be pneumonia), presumed malaria (based on fever) or diarrhoea. This training included identification of danger signs and when to refer severe cases. Sessions to demonstrate some difficult concepts like fast breathing were held in clinical settings at health centres or hospital level. After completion of training each volunteer was provided with drugs (ACT for fever, ORS and zinc for diarrhoea, and Amoxicillin for ARI), respiratory timers, job aids (algorithms for diagnosis and treatment), and registers to record treatments. A total of 11,170 VHT members were trained in the VHT basic package for six initial days. The iCCM training thereafter was conducted among 5,585 VHT members for another five days across the eight districts.

Supplies were purchased by UNICEF and distributed to each district by Malaria Consortium staff. Health facility workers were trained to supervise VHTs, summarize and report compiled data, and to inform patients of the availability of VHTs. VHTs were supervised by health facility and Malaria Consortium staff, as well as their peer supervisors in each designated parish. Supervision consisted of home visits conducted by health workers and quarterly meetings. VHTs were provided with transport refund and a meal during quarterly meetings and treatment supplies were replenished during these meetings. The attrition rate was low, only 3.8%. Community mobilization activities included radio spots announcing the importance of seeking care for the three conditions and availability of VHTs. Community leaders were trained to sensitize communities about the work of VHTs.

Three comparison districts were selected based on the following criteria: 1) no current or planned iCCM programme in the near future and 2) similar socioeconomic and demographic profile to the intervention area based on the most recent census (2002), Demographic and Health Survey (2006) and Malaria Indicator Survey (2009) results. The three selected districts were Luwero, Nakaseke and Rakai which had a combined 2012 population of 1,115,700. In some districts (Luweero, Nakaseke) there existed a semi-functional VHT system with some sub-counties having VHTs trained in the MOH’s basic VHT package by Plan International and World Vision International, but none of the VHTs in the comparison area were trained in iCCM and none provided treatments.

### Data Sources

#### Routine and Contextual Data

VHTs reported on availability of commodities and treatments given on a monthly basis using standardized registers. Peer-supervisors summarized VHT data and sent it to the respective health facility affiliated with the parish. The reports were then sent to the district health management information systems (HMIS) focal person and Malaria Consortium. Facility treatment data were also collected from the HMIS in both the intervention and comparison districts. Data on health programs taking place in the intervention and comparison districts during the study period were obtained from district officials in a standardized form. Relevant contextual factors, such as national stock outs of medicines, or disease outbreaks, were documented.

#### Household Surveys

The household survey was conducted at baseline, before implementation of iCCM (October 2010) and at end line, after two years of implementation in October 2012 for the intervention area, and was delayed to February 2013 in the comparison area due to an Ebola outbreak. The survey was done using a two stage cluster sampling methodology. For the baseline survey, first 40 clusters (enumeration areas [EA]) were selected using the census data obtained in 2002 with probability proportionate to size technique. Second, all households within the selected clusters were mapped and 26 households were selected based on simple random sampling from a computer-generated table of random numbers. At end line, the two-stage cluster sampling procedure was repeated but this time 100 clusters were selected with 40 households within each cluster to increase the sample size in order to obtain a mortality estimate based on birth histories. Leaders of each district and community within the selected EA were sensitized about the upcoming survey over a period of 14 days prior to the surveys. All interviewers in both the intervention and comparison areas participated in a two-day training with a mock interview exam at the end of the training to test their ability to complete the relevant questionnaires. Only those who passed the exam were chosen to be interviewers. The interview teams consisted of a supervisor and 6 interviewers.

There were three questionnaires.(S1 Excel Spreadsheet) The first administered to the head of the household and collected data on socio‐demographic and economic characteristics of the household. The second on child health was administered to the primary care taker of children under 5 years of age and collected data on symptoms of fever, diarrhoea, or ARI (indicated by cough and fast breathing), care seeking and treatments. The third collected data on women of reproductive age (15–49 years of age) and included education levels attained and birth history. Questionnaires were pretested in Kiboga another iCCM implementing district not part of this study. The household head provided consent for the household to participate in the interview. In addition, individual informed consent was sought from all other respondents before interviews were conducted, captured either by the signature of the respondent or, if they were illiterate, by their right hand thumbprint. Before each interviewee was asked to give consent, the interviewer gave a brief description of the survey objectives, the data collection procedure, the potential harm to participants, the expected benefits, and the voluntary nature of participation at all stages of the interview. Consent forms were collected and secured in a locked file with only access by the lead principle investigator. All participants were ensured that data would be kept confidential and would not be shared with non-project staff.

### Ethics

The Uganda National Council for Science and Technology approved this study on November 22, 2010.

### Outcomes

The primary outcome was coverage defined as the proportion of children with fever, ARI (cough and fast breathing) or diarrhoea in the past 2 weeks who were brought to health care and received appropriate treatment (either ACT, Amoxicillin, ORS and zinc). Health care seeking rates and timeliness (defined as within 24 hours of symptoms) of health care seeking, and treatment, and under-five mortality were also examined in the intervention and comparison areas.

### Statistical analysis

Routine reporting data were double entered into Excel. Descriptive analysis reporting on number of treatments over time among VHTs and facilities was done. For the iCCM program, the average across a year of the proportion of VHTs receiving a supervision visit and having stock outs each quarter was calculated.

The baseline household survey sample included 40 clusters (villages) of 26 households (n = 1,040) in both the intervention and comparison areas, (2,080 households). At end line the sample was increased to 100 clusters of 40 households (n = 4,000) in both the intervention and comparison (8,000 households).

For the household survey, proportions, odds ratios (ORs) and 95% confidence intervals (CI) were calculated from bivariate and multivariable analyses, weighted to account for the complex survey design and non-response. Proportions in the intervention and comparison groups were compared at baseline and end line using a two-sided chi-square test. A difference-in-differences (DID) analysis was done to study whether outcomes of interest were significantly different between groups overtime. This was done using a multivariable logistic regression model that included group, time and an interaction term of group with time in the model. The model also controlled for socioeconomic and demographic differences between intervention and comparison areas, except for rural urban residence as that was co-linear with wealth status. A significant coefficient of the interaction term implies that the outcomes differed by groups over time. The odds ratios were calculated using the difference in the comparison group as the reference. A two-sided p-value <0.05 was considered to be significant for all tests. Data analysis was done using [12] and EpiData 3.1 [13] (household survey data). Household survey data entry also included consistency checks and automated skip patterns. (S1 Table, S1 Dataset)

### Modelling mortality impact

Lives Saved Tool (LiST) was used to estimate the number of lives saved and mortality impact [14]. LiST is based on a linear, mathematical model that is deterministic [15]. The relationship between changes in intervention coverage and one or more outputs (e.g., cause specific mortality, lives saved) are specified in terms of the effectiveness of the intervention for reducing the probability of that outcome. Outputs include absolute number of lives saved (and, if a negative number, lives lost) and cause of death. To compare the intervention and comparison areas a separate projection was created for each area using baseline and end line surveys.

Using information from the 2010 Uganda Census the population sizes for each area were estimated and entered into LiST. The actual ARI, malaria, diarrhoea treatment, immunization and bed net coverage data for 2010 and for 2012 based on the surveys were entered while coverage data for 2011 were based on a linear extrapolation. For all other interventions the results of 2011 Demographic and Health Survey, Central region was used [16]. These later coverage data were entered for 2010 and kept unchanged in 2011 and 2012. Finally, based on the birth history conducted in the 2012 household surveys; the under-five mortality rates for 2010 were calculated and these rates were used for each area in the model. The lives saved, under-five mortality rates, and causes of death in the intervention and comparison areas from 2010 to 2012 were then modeled.

## Results

### Contextual Factors

There were frequent national level amoxicillin stock-outs throughout the implementation period.

Zinc was not on Uganda’s Essential Drug list until October 2011.

Early on in the project, the intervention area districts were split by the government creating eight districts from the original three districts. In the intervention area, there were 23 other health programs, which included two malaria-specific programs, and none providing community based treatments.

In the comparison area, there were 36 health programs, of which only one program was malaria specific and none provided community based treatments. The comparison area also experienced an Ebola outbreak last quarter of 2013, which delayed the end line household survey until February 2014. In addition, due to the outbreak there were extensive preventive activities focusing on hygiene (e.g. hand washing) in the comparison area just prior to the survey.

### Routine Reporting Results

Between July 2010 and December 2012, a total of 5,586 VHTs were trained in iCCM. In 2012, 96% were still active. Supervision rates were high; on average, 80% of VHTs were supervised quarterly (Table 1). Except for amoxicillin, stock outs were relatively minimal. By the end of 2012, 1,907,746 treatments had been provided by VHTs. Although facility-based treatments in the intervention area decreased during this time period, overall treatments increased by 20%. In the comparison area, facility-based treatments for the three conditions increased by 9% (Table 1).

**Table 1:** Information on program implementation and treatments from routine reporting from Village Health Teams and Health Facilities, Central Uganda Integrated Community Case Management Program, 2011–2012.

	2011		2012	
Reported data	Intervention	Comparison	Intervention	Comparison
Proportion of VHT members supervised quarterly	75%	NA	94%	NA
Proportion of VHT members without stock-out of ACT	76%	NA	84%	NA
Proportion of VHT Members without stock-out of ORS^3^	96%	NA	98%	NA
Proportion of VHT without stock-out of zinc	NA	NA	84%	NA
Proportion of VHT Members without stock-out of amoxicillin	61%	NA	61%	NA
VHT Based Malaria Treatments (ACT)	352,202	NA	922,046	NA
Facility Based Malaria Treatments (ACT)	840,107	267,619	386,996	287,957
VHT Based Diarrhoea Treatments (ORS and zinc)	64,971	NA	109,459	
Facility Based Diarrhoea Treatments (ORS and zinc)	69,161	22,019	61,023	28,425
VHT Based Acute Respiratory Illness Treatments (amoxicillin)	102,612	NA	356,456	
Facility Based Acute Respiratory Illness Treatments (amoxicillin)	82,848	22,915	70,194	25,802
Total VHT Based Treatments	519,785		1,387,961	
Total Facility-Based Treatments	992,116	312,553	518,213	342,184
Total reported treatments	1,511,901	312,553	1,906,174	342,184

VHT, Village Health Team; ACT, Artemisinin-based combination therapies; ORS, Oral Rehydration Solution

### Household Survey Results

The survey response rate was high: 99% (2076/2080) and 97% (7,734/8000) of eligible households participated at baseline and end line, respectively. The intervention and comparison areas differed in the proportion living in rural areas, the proportion of households with children under five, household size, age of head of household and education of caretaker (Table 2).

**Table 2:** Household Survey, Distribution of characteristics by study group, Integrated Community Case Management, Central Uganda, 2010 and 2012.

Characteristic	Baseline (2010)			End line (2012)		
					
	Intervention	Comparison		Intervention	Comparision	
	(N = 1036)	(N = 1,040)		(N = 2,012)	(N = 1,957)	
	% (95%CI)	% (95%CI)	P-value	% (95%CI)	% (95%CI)	P-value
Location						
Rural	67.5 (64.6–70.4)	87.5 (85.5–89.5)	<0.001	73.6 (72–74.9)	87.0 (86.0–88.1)	<0.001
Urban	32.5 (29.6–35.5)	12.5 (10.5–14.5)	<0.001	26.4 (25.0–27.8)	13.0 (10.1–14.9)	<0.001
Age of household head						
18–24 years	7.5 (5.5–10.2)	6.2 (4.7–8.2)	0.197	4.2 (3.3–5.3)	5.5 (4.5–6.9)	0.081
25–29 years	17.2 (13.6–21.4)	15.1 (12.7–17.8)	0.212	10.3 (8.8–12.0)	13.2 (11.4–15.3)	0.010
30–34 years	17.6 (14.7–20.9)	18.0 (15.6–20.6)	0.512	15.1 (13.3–16.9)	18.5 (16.4–20.7)	0.412
35–39 years	18.9 (15.8–22.4)	20.4 (18.2–22.8)	0.635	17.8 (15.8–20.0)	18.9 (16.8–21.1)	0.223
40–44 years	13.0 (10.3–16.2)	12.5 (10.5–14.8)	0.382	10.4 (9.0–12.0)	11.4 (10.0–12.9)	0.072
45–49 years	7.5 (5.8–9.5)	8.5 (6.7–10.7)	0.210	8.4 (7.1–10.0)	8.3 (6.9–9.9)	0.056
50+ years	18.4 (14.9–22.6)	19.4 (16.9–22.2)	0.130	33.9 (30.8–37.1)	24.2 (21.7–26.9)	0.004
Average Household size	5.66 (5.42–6.91)	6.02 (5.84,6.20)	<0.001	5.84 (5.70,5.96)	5.07 (4.96,5.19)	<0.001
Household wealth rank^a^						
Poorest	27.5 (20.2–36.4)	39.1 (31.6–47.0)	0.104	30.4 (26.7–34.5)	36.3 (32.5–40.4)	0.102
Poor	29.3 (24.1–35.1)	37.3 (33.2–41.7)	0.312	31.8 (29.0–34.8)	34.8 (32.3–37.3)	0.150
Least Poor	43.2 (32.9–54.0)	23.6 (16.7–32.2)	0.041	37.7 (32.0–43.8)	28.9 (24.1–34.3)	0.211
Number of children in sample	1,479	1,591	-	2,356	2,631	-
Child’s age (years)						
<1	19.1 (17.1–21.1)	17.6 (16.1–19.1)	0.241	33.9 (31.6–36.2)	18 (16.5–19.5)	<0.001
1	18.6 (16.6–20.6)	16.4 (14.9–17.9)	0.079	22.1 (20.1–24.1)	21.3 (19.7–22.9)	0.54
2	18.1 (16.1–20.1)	18.5 (16.9–20.1)	0.755	14.0 (12.3–15.7)	17.6 (16.1–19.1)	0.002
3	21.8 (19.7–23.9)	22.8 (21.1–24.5)	0.470	14.6 (12.9–16.3)	22.5 (20.9–24.1)	<0.001
4	22.3 (20.8–24.4)	24.7 (23.0–26.4)	0.089	15.5 (13.7–17.3)	20.6 (19.1–22.1)	<0.001
Education level of care taker						
No School	13.1 (9.8,17.4)	18.5 (14.4–23.4)	0.001	14.5 (12.0,17.5)	14.7 (12.4,17.3)	0.829
Education Level for those who attended school						
Primary	54.9 (48.2,61.4)	70.3 (64.6,75.5)	<0.001	61.4 (6.9,65.8)	62.6 (58.4,66.6)	0.345
“O” level–Middle School	36.6 (31.9,41.8)	26.7 (22.2–31.7)	<0.001	32.2 (28.4,36.3)	30.7 (27.7,34.0)	0.218
“A” level–High School	3.8 (2.5–5.8)	1.1 (0.6,1.9)	<0.001	2.4 (1.6,3.5)	2.9 (2.1–4.0)	0.232
University/Tertiary	4.7 (3.1–7.0)	1.9 (1.1,3.2)	0.049	4.0 (2.8–5.6)	3.7 (2.7–5.2)	0.553

CI, Confidence Interval

a. ‘Poorest’ and ‘Least Poor’ defined by lowest and highest wealth quintiles based on principal components analysis of household assets.

### Fever

The proportion of children under five that were reported to have had fever in the last two weeks in the intervention area did not change significantly from baseline to end line, but decreased in the comparisons area although the DID was not significant (Table 3).

**Table 3:** Disease prevalence, care seeking and treatment coverage among children U5 at baseline and end line by study group, Integrated Community Case Management Central Uganda 2010 and 2012.

Measure	Intervention			Comparison			DID estimator ^a^	
	Baseline	End line		Baseline	End line			
	N = 1,479	N = 2,356		N = 1591	N = 2576			
	%(95%CI)	%(95%CI)	p-value	%(95%CI)	%(95%CI)	p-value	AORa (95%CI)	p-value
***Fever***								
Prevalence	16.1(12.9,20.0)	15.6(13.5,17.9)	0.562	23.5(20.1,26.9)	19.6(17.4,22.0)	0.014	1.12(0.71,0.77)	0.621
Sought care	75.9(69.6–81.3)	91.8 (88.2,94.4)	<0.001	87.1(82.4, 90.6)	90.8(87.5,93.2)	0.142	2.36(1.1,5.09)	0.028
Care seeking in 24 hours	35.0 (28.1,42.6)	56.0 (49.7,62.0)	<0.001	43.1(36.2,50.4)	53.8(48.3,59.2)	0.030	1.60(0.86,2.97)	0.136
Received ACT	32.5(26.2,39.5)	64.3(57.9,70.2)	<0.001	49.3(43.7,55.0)	67.7(61.9,72.9)	<0.001	1.57(0.91,2.70)	0.105
Took ACT within 24 hours of symptoms	19.4(14.1,26.2)	44.7(39.1,50.4)	<0.001	35.0(29.4,41.1)	47.5(42.3,52.8)	0.017	2.12(1.08,4.14)	0.029
***Acute Respiratory Illness***								
Prevalence	12.3(9.5,15.8)	12.1(10.1,14.3)	0.100	18.6(16.1,21.4)	15.0(13.3,16.8)	0.015	1.17(0.82,1.66)	0.389
Sought care	55.5 (45.0,65.6)	76.4 (67.9–83.2)	<0.001	80.1(70.6,87.0)	67.0(86.2,92.3)	0.015	6.06(2.79,13.15)	<0.001
Care seeking within 24 hours of symptoms	13.2(8.5,19.8)	43.3(35.8,51.0)	<0.001	25.2(19.1,32.3)	33.9(28.3,39.8)	0.063	3.95(1.83,8.54)	<0.001
Received Antibiotic	38.3(29.3,48.1)	28.8(22.2,36.4)	0.141	67.0(58.9,74.1)	19.0(14.5,24.6)	<0.001	5.18(2.32,11.57)	<0.001
Took antibiotic within 24 hours of symptoms	8.8(4.9,15.4)	13.5(8.7,20.4)	0.212	19.0(13.6,25.9)	6.2(3.9,9.7)	<0.001	7.95(2.57,24.62)	<0.001
***Diarrhoea***								
Prevalence	9.2(6.9,12.0)	7.9(6.8,9.3)	0.499	12.6 (10.4–15.1)	7.3 (6.1,8.7)	<0.001	1.60(1.06,2.42)	0.027
Sought care	43.3(32.0,55.3)	59.4(50.4,67.8)	0.034	69.7(60.4,73.8)	55.9(47.9,63.6)	0.031	2.55(1.04,6.27)	0.042
Received ORS and zinc	2.2(0.8,6.1)	15.9(11.3,21.1)	<0.001	5.5(2.4,11.1)	1.7(0.8,5.6)	0.142	22.9(3.66,142.99)	0.001

CI, Confidence Interval; DID, differences of differences; CI, confidence interval; ACT, Artemisinin-based combination therapies; AOR, Adjusted Odds Ratio

a. Reference is comparison group difference overtime and is adjusted for differences in child’s age, education of caretaker, household size and wealth and age of household head

In the intervention area, the proportion of children under five with a fever who sought care within 24 hours improved from 35.0% at baseline to 56.0% at end line. Among them, the proportion that received ACT within 24 hours of symptoms increased from 19.4% to 44.7%. Although there were increases in the comparison area these increases were smaller than those in the intervention area. The DID analysis showed significant improvements in care-seeking and receipt of ACT within 24 hours in the intervention area (Table 3).

**Fig 1:**
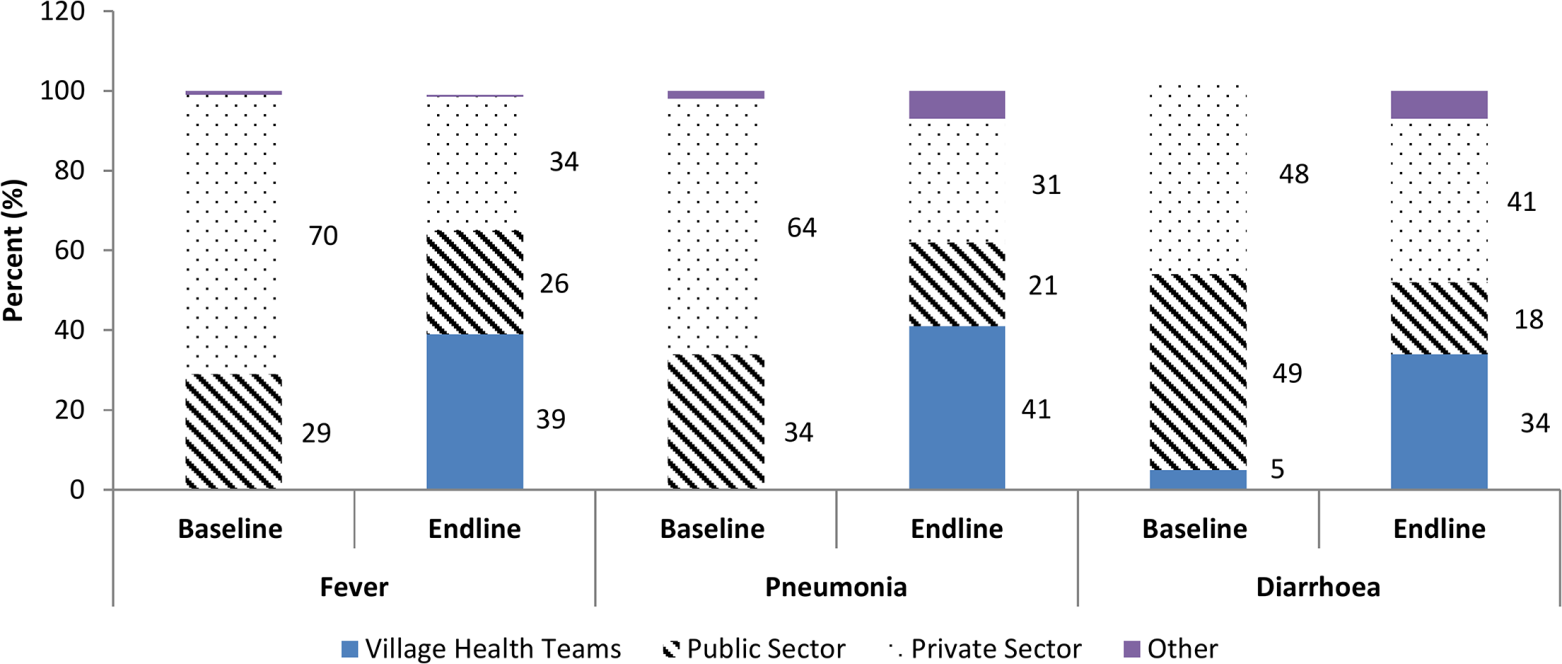
Sources of treatment for children with fever, acute respiratory illness and diarrhoea in intervention area comparing baseline to end line, Uganda 2010–2012.

In the intervention area, of caregivers who sought treatment for their child with fever, there was a sharp increase in those who sought care from a VHT from 0% at baseline to 39% at end line, while facility-based care usage decreased from 99% to 60%. (Fig 1).

### ARI/ Presumed Pneumonia

The proportion of children under five reported to have had ARI in the last two weeks in the intervention area did not differ significantly from baseline to end line, but did decrease in the comparison, although the DID was not significant (Table 3).

In the intervention area, healthcare seeking for ARI within 24 hours of symptom onset increased significantly, but the proportion of those who received an antibiotic after seeking care declined from 38.3% to 28.8%. Among those who sought care, the proportion that received an antibiotic within 24 hours increased significantly (Table 3). In the comparison area, the proportion that sought health care decreased significantly, there were large significant decreases in the proportion of children who received any antibiotic and, among those, who received an antibiotic within 24 hours of symptom onset. The DID analysis showed significant improvements in care seeking, care seeking within 24 hours, and receipt of antibiotics within 24 hours, and a significantly smaller decline in antibiotic coverage in the intervention area.

In the intervention area, of those caregivers who sought treatment for an ARI, the proportion who sought care from a VHT increased from 0% to 41%, while facility-based care usage (public or private) decreased from 98% to 52% (Fig 1).

At baseline in the intervention area, a larger proportion of the children with an ARI received Cotrimoxazole (26.5%) compared to those that received amoxicillin (17.4%). At end line, only

11.7% were given Cotrimoxazole while 33.3% were given amoxicillin.

### Diarrhoea

The proportion of children under five that were reported to have had diarrhoea in the last two weeks decreased insignificantly from 9.2% to 7.9% in the intervention and signiciantly from 12.6% to 7.3% in the comparison areas. The DID was significant (Table 3).

In the intervention area, the proportion of caregivers of children under five with diarrhoea who sought any care increased from 43.3% to 59.4%. The proportion of those with diarrhoea that received ORS and zinc increased significantly (Table 3). In contrast, in the comparison area treatment seeking for children under five with diarrhoea declined and the proportion that received both ORS and zinc together declined. The DID analysis showed significant improvements in care seeking and treatment with both ORS and zinc in the intervention area.

In the intervention area, of all caregivers who sought treatment for their child with diarrhoea, the proportion of those who went to a VHT increased from 5% to 34%, while facility based care usage decreased from 97% to 59% (Fig 1).

### LiST Results

The estimated under-five mortality in the intervention area slightly decreased from 50 deaths per 1000 live births to 49. However, mortality in the comparison arm increased from 63 to 69 deaths per 1000 live births. In addition, the model indicates that 106 lives of children under five were saved the intervention area, whereas in the comparison area 311 lives of children were lost. In both the intervention and comparison area the top causes of death were neonatal in 2010 and again in 2012. In 2010, 28% of deaths in the intervention area were due to pneumonia, malaria or diarrheoa; in the comparison area, 32% were due to ARI, malaria and diarrhoea. At the end of the study period in 2012, there was a slight decrease in the proportion (26%) of deaths due to ARI, malaria, and diarrhoea in the intervention area. However, in the comparison area there was an increase in the proportion of deaths (38%) due to ARI, malaria and diarrhea. Among the lives saved in the intervention area, 57% was due to antimalarial treatment, 21% was due to treatment with ORS and zinc, 9% was due to use of insecticide treated nets, and the remaining 20% was due to other maternal, newborn, and child health interventions.

## Discussion

There have been a limited number of studies that have shown a positive impact of community treatment of pneumonia and malaria in Sub-Saharan Africa. Our findings show that iCCM contributed to significant increases in treatment coverage for diarrhoea, in health care seeking for all three diseases and timeliness of treatment for malaria and ARI. In addition, it mitigated a decline in overall treatment coverage for ARI that may have been due to national stock outs of amoxicillin. Finally our modelling suggests that lives were saved in areas with iCCM while lives were lost in the area without iCCM.

There is growing evidence of the quality of treatment that CHWs in Sub-Saharan Africa can provide [17–20]. In Uganda, one study found that caregivers may first seek care for a sick child from drug shops, but the quality of care received was sub-standard [21]. In that same area VHTs trained to deliver pneumonia and malaria treatments provided high quality services and were accessed more often by caretakers [22]. Other studies in Uganda found using VHTs resulted in high rates of appropriate treatment of pneumonia symptoms and high adherence to both antimalarial and antibiotics [23, 24]. Also VHTs correctly used RDTs for malaria diagnosis leading to appropriate use of ACTs [25].

The study also found evidence of high quality services provided by VHTs such as high supervision rates and increased use of amoxicillin for ARI treatment as opposed to other antibiotics. Of particular note is the increased timeliness of malaria and pneumonia treatments, which are critical to prevent severe illness and mortality [2, 26]. Using VHTs to provide treatments closer to the community facilitated such timely treatments.

VHTs may have also eased the burden on facilities. In the intervention area, the facility based treatment numbers decreased while the number of treatments dispensed by VHTs increased substantially. This suggests that VHTs were seeing some patients that may have otherwise gone to facilities but given that overall number of treatments from both sources increased, VHTs were also providing for unmet demand. This was also found in a similar study conducted in Sierra Leone that, unlike Uganda, did not show a statistically significant increase in coverage in the intervention area, but did show decreased reliance on facilities as coverage increased [27].

Although there was a significant increase in the use of both ORS and zinc for diarrhoea in the intervention area, the overall proportion at end line was just over 15%. The fact that zinc was not on the essential drug list until October 2011 may have contributed to this. However, there may have been incorrect dispensing practices of giving ORS without zinc even when it was available. Finally, care givers are less likely to seek care for diarrhoea which has been demonstrated in studies in other countries [3, 28].

Measuring mortality impact of any health program in sub-Saharan Africa is difficult for multiple reasons including lack of civil vital registration, difficulty ensuring the quality of birth history data collected to calculate childhood mortality rates, and the large sample sizes required to detect changes in mortality rates. A recent review of eight iCCM studies that attempted to measure mortality impact found six studies that showed decline in mortality but only one demonstrated a significant decline partly due to reasons described above but also because in some cases mortality was measured prior to full scale up of the programs [29]. Given these issues it is recommended to model the impact of iCCM [30, 31]. For this study, based on LiST, after only two years of implementation, lives were saved in the area with iCCM while lives were lost in the area without iCCM.

There were some limitations to our study. The demographics of the comparison area were slightly different than the intervention area; however, this was controlled for in the DID analysis. Reported information on morbidity in the household survey is subjective; it relied on the caregiver’s perception of illness and treatments provided. The accuracy of the routine data was not verified. Data on timeliness of diarrhoea treatment were not collected. Data collection for the household survey at end line was done at a different time in the comparison area, during the dry season, then the intervention area, during the wet season, which may explain the decline in prevalence found in the comparison area. In addition, prevention activities in the comparison area after the Ebola outbreak may have caused the large decrease prevalence of diarrhoea found.

## Conclusion

This study demonstrated that iCCM significantly increased treatment coverage for children U5 afflicted with fever and diarrheoa, improved timeliness of treatments for those children with fever and mitigated the effect of national stock outs of amoxicillin on ARI treatment. In addition, high quality treatment was provided at the community level and the treatment burden at health facilities was reduced. Finally, lives were saved in the areas with iCCM (mostly attributable to ORS and zinc and antimalarial treatment) and lost in the areas without iCCM. Continued support and expansion of iCCM in Uganda could contribute significantly to the reduction in U5 deaths.

## Supporting Information

S1 Dataset Stata Minimal Dataset of Integrated Community Case Management in Central Uganda. http://dx.doi.org/10.6084/m9.figshare.1460836
(XLS)

S1 Excel Spreadsheet Consent and Questionnaire for household survey of Integrated Commuity Case Management in Central Uganda. http://dx.doi.org/10.6084/m9.figshare.1460838
(XLSX)

S1 Table Data Dictionary for minimal dataset of Intergreated Commuity Case Management in Central Uganda. http://dx.doi.org/10.6084/m9.figshare.1460837
(DOCX)
