# Supplementary material for: Evaluation of Integrated Community Case Management in Eight Districts of Central Uganda
Source: PLoS One. 2015 Aug 12;10(8):e0134767. doi: 10.1371/journal.pone.0134767 (PMC4534192; doi:10.1371/journal.pone.0134767)
Supplement: S1 Table — http://dx.doi.org/10.6084/m9.figshare.1460837 (DOCX) [file pone.0134767.s003.docx]

**Data dictionary for the iccm_uganda_childhealth_min_dataset**

| **Variable name** | **Value and meaning** |
| --- | --- |
| survey | 0 = Baseline |
|  | 1 = Endline |
| arm | 0 = Comparison |
|  | 1 = Intervention |
| hhid | Household ID: Uniquely identifies HH uniquely |
| childid | Child ID: Uniquely identifies child uniquely |
| interaction | Interaction term between survey and arm |
| quintile | 3 quantiles of the ses (wealth proxy) |
| agehh | age of the HH head |
| sexhh | sex of HH head |
| hhsize | HH size |
| education | Education level of caretaker |
|  | 0=No education |
|  | 1=Primary |
|  | 2=Secondary O'level |
|  | 3=Secondary A'level |
|  | 4=University / Tertiary |
| cough | Has child had cough in the last 2 weeks |
|  | 1=Yes |
|  | 2=No |
|  | 8=Don't Know |
| fastbrea | Has child had fast breathing in the last 2 weeks |
|  | 1=Yes |
|  | 2=No |
|  | 8=Don't Know |
| seekcough | Sought treatment for cough |
|  | 1=Yes |
|  | 2=No |
| treat1c | *1st treatment given for cough* |
|  | A = NOTHING |
|  | B = ASPIRIN |
|  | C = PANADOL |
|  | D = AMOXYCILLIN |
|  | E = ERYTHROMYCIN |
|  | F = AZYTHROMYCIN |
|  | G = SEPTRIN |
|  | X = OTHER |
|  | Z = DON'T KNOW |
| treat2c | *2nd treatment given for cough* |
|  | Same as treat1c codes above |
| treat3c | *3rd treatment given for cough* |
|  | Same as treat1c codes above |
| othtrtc | Other treatment given for cough |
| source1c | Source of first treatment for cough |
|  | **PUBLIC MEDICAL SECTOR** |
|  | A = HOSPITAL |
|  | B = HEALTH CENTER III |
|  | C = HEALTH CENTER II |
|  | D = CLINIC/OUTREACH |
|  | E = VILLAGE HEALTH TEAM/CMD |
|  | F = OTHER PUBLIC |
|  | **PRIVATE MEDICAL SECTOR** |
|  | G = PVT. HOSPITAL/ CLINIC |
|  | H = PHARMACY/ DRUG SHOP |
|  | I = PVT DOCTOR |
|  | J = CLINIC/OUTREACH SERVICES |
|  | K = COMMUNITY HEALTH WORKER |
|  | L = OTHER PRIVATE |
|  | **OTHER SOURCE** |
|  | M = SHOP |
|  | N = TRADITIONAL PRACTIONER |
|  | X = OTHER |
| source2c | *Source of second treatment for cough* |
|  | Same as source1c codes above |
| source3c | *Source of third treatment for cough* |
|  | Same as source1c codes above |
| othpubc | Other Public source |
| othpvtc | Other Private source |
| othsrcc | Other source source in the Other category |
| src1stc | 1st source of treatment for cough |
|  | Can take on any of the codes from source1c above |
| timetrtc | days to treatment of cough |
|  | **Range is 0 - …. 98 = Not Given** |
| fever | Child had fever in the last 2 weeks |
|  | 1=Yes |
|  | 2=No |
|  | 8=Don't Know |
| fstart | days ago fever started |
|  | **Range is 0 - …. 98 = Not Given** |
| seekfever | Sought treatment for fever |
|  | 1=Yes |
|  | 2=No |
| source1f | Source of first treatment for fever |
|  | PUBLIC MEDICAL SECTOR |
|  | A = HOSPITAL |
|  | B = HEALTH CENTER III |
|  | C = HEALTH CENTER II |
|  | D = CLINIC/OUTREACH |
|  | E = VILLAGE HEALTH TEAM/CMD |
|  | F = OTHER PUBLIC |
|  | PRIVATE MEDICAL SECTOR |
|  | G = PVT. HOSPITAL/ CLINIC |
|  | H = PHARMACY/ DRUG SHOP |
|  | I = PVT DOCTOR |
|  | J = CLINIC/OUTREACH SERVICES |
|  | K = COMMUNITY HEALTH WORKER |
|  | L = OTHER PRIVATE |
|  | OTHER SOURCE |
|  | M = SHOP |
|  | N = TRADITIONAL PRACTIONER |
|  | X = OTHER |
| source2f | *Source of second treatment for fever* |
|  | Same as source1f codes above |
| source3f | *Source of third treatment for fever* |
|  | Same as source1f codes above |
| othpubf | Other Public source |
| othpvtf | Other Private source |
| othsrcf | Other source source in the Other category |
| src1stf | 1st source of treatment for cough |
|  | Can take on any of the codes from source1c above |
| timetrtf | days to treatment of cough |
|  | **Range is 0 - …. 98 = Not Given** |
| drugsf | Was Child given drugs for fever treatment |
|  | 1=Yes |
|  | 2=No |
| al | Was Child given AL (coartem) |
|  | 1=Yes |
|  | 2=No |
| asaq | Was Child given AS+AQ (Artesunate + Amodiaquin) |
|  | 1=Yes |
|  | 2=No |
| assp | Was Child given AS+SP (Artesunate + Fansidar) |
|  | 1=Yes |
|  | 2=No |
| timeal | How many days after the fever started did child first take AL |
|  | 0 = Same day |
|  | 1 = Next day |
|  | 2 = Two days after fever |
|  | 3 = Three days after fever |
|  | 4 = Four days or more after fever |
|  | 8 = Don't know |
| ndal | For how many days did child take AL |
|  | 0 ….., 8 = Don't Know |
| srcal | Source of AL |
|  | Same as source1f codes above |
| othsrcal | Other source of AL |
| timeasaq | How many days after the fever started did child first take AS + AQ |
|  | 0 = Same day |
|  | 1 = Next day |
|  | 2 = Two days after fever |
|  | 3 = Three days after fever |
|  | 4 = Four days or more after fever |
| ndasaq | For how many days did child take AS + AQ |
|  | 0 ….., 8 = Don't Know |
| srcasaq | Source of AS + AQ |
|  | Same as source1f codes above |
| othsrcassp | Other source of AS + AQ |
| timeassp | How many days after the fever started did child first take AS + SP |
|  | 0 = Same day |
|  | 1 = Next day |
|  | 2 = Two days after fever |
|  | 3 = Three days after fever |
|  | 4 = Four days or more after fever |
| ndassp | For how many days did child take AS + SP |
|  | 0 ….., 8 = Don't Know |
| srcassp | Source of AS + SP |
|  | Same as source1f codes above |
| othsrcassp | Other source of AS + SP |
| diarrhea | Child had diarrhoea in the last 2 weeks |
|  | 1=Yes |
|  | 2=No |
|  | 8=Don't Know |
| seekdiarr | Sought treatment for diarrhoea |
|  | 1=Yes |
|  | 2=No |
| homefluid | Was given homefluid for treatment of diarrhoea |
|  | 1=Yes |
|  | 2=No |
|  | 8=Don't Know |
| ors | Was given ors for treatment of diarrhoea |
|  | 1=Yes |
|  | 2=No |
|  | 8=Don't Know |
| treat1d | What was child given to treat diarrhoea (first drug mentioned) |
|  | A=ZINC |
|  | B=PILL OR SYRUP |
|  | C=INJECTION |
|  | D=(I.V.) INTRAVENOUS |
|  | E=HOME REMEDIES/HERBAL MEDICINES |
|  | X=OTHER |
| treat2d | What was child given to treat diarrhoea (second drug mentioned) |
|  | A=ZINC |
|  | B=PILL OR SYRUP |
|  | C=INJECTION |
|  | D=(I.V.) INTRAVENOUS |
|  | E=HOME REMEDIES/HERBAL MEDICINES |
|  | X=OTHER |
| treat3d | What was child given to treat diarrhoea (third drug mentioned) |
|  | A=ZINC |
|  | B=PILL OR SYRUP |
|  | C=INJECTION |
|  | D=(I.V.) INTRAVENOUS |
|  | E=HOME REMEDIES/HERBAL MEDICINES |
|  | X=OTHER |
| othtrtd | Other drug given to treat diarrhoea |
